# Teaching and Learning with Mobile Technology: A Qualitative Explorative Study about the Introduction of Tablet Devices in Secondary Education

**DOI:** 10.1371/journal.pone.0144008

**Published:** 2015-12-07

**Authors:** Hannelore Montrieux, Ruben Vanderlinde, Tammy Schellens, Lieven De Marez

**Affiliations:** 1 Department of Educational Studies, Ghent University, Gent, Belgium; 2 iMinds-MICT-Department of Communication Sciences, Ghent University, Gent, Belgium; University of Westminster, UNITED KINGDOM

## Abstract

This paper investigates teachers’ and students’ perceptions concerning the impact of using tablet devices for teaching and learning purposes. An explorative focus group study was conducted with teachers (n = 18) and students (n = 39) in a secondary school that has implemented tablet devices since 2012. The general finding of this study shows that the use of tablet devices in the classroom setting has an impact on both teaching and learning practices. The results suggest that teachers can be divided into two categories: the innovative teachers and the instrumental teachers. Innovative teachers attempt to shift from a teacher-centered to a learning-centered approach. They have changed their teaching style by transforming lessons in accordance with the advantages tablet computers can offer. Instrumental teachers seem to use the device as a ‘book behind glass’. The distinction between the two groups has consequences for both the way courses are given and how students experience them. In general, the introduction of tablet devices entails a shift in the way students learn, as the devices provide interactive, media-rich, and exciting new environments. The results of this study indicate that policy makers should consider introducing technical and pedagogical support in order to facilitate both teachers’ and students’ understanding of the full potential of this kind of technology in education.

## Introduction

Information and communication technology is a principal driver in our Information Society [1] of which the immediate consequences for educational practice can be observed [2]. Following this evolution, several authors [3,4] have mentioned the need to shift from the traditional classroom setting, where the student is seen as a passive consumer of educational knowledge, to a classroom in which learners are considered active participants and where collaboration and sharing information in a resource-rich environment is given precedence. To advance this shift and the necessary educational reform, hardware and software developers promote new technological tools, and more specifically tablet devices, as magic devices [5,6]. These tablets are more narrowly defined by the New Media Consortium in 2012 as iPads, Windows -or Android devices, i.e. small, wireless, mobile personal computers which have finger-driven touch screens and are backed-up by diverse applications in a well-provisioned application marketplace [7].

According to this transformation to a more technology-enhanced learning approach, Hattie [8] has indicated that: *"An analysis of the meta analyses of computers in schools indicates that computers are used effectively (a) when there is a diversity of teaching strategies; (b) when there is a pre-training in the use of computers as a teaching and learning tools; (c) when there are multiple opportunities for learning (e*.*g*. *deliberative practice*, *increasing time on task); (d) when a student*, *not teacher*, *is in "control" of learning; (e) when peer learning is optimized; and (f) when feedback is optimized*.*"* [8]. In other words, Hattie [8] claimed that the following conditions should be fulfilled in order to integrate technology into the classroom; namely the role of the teacher, the need of professionalization, and the need of adapted teaching and learning approaches.

While it can be argued that the use of technology during classes can support constructivist approaches [9,10], implementing technology into classes does not imply a radical change of the didactics [11,12]. According to Yelland [13] learning with technology needs more than making learning activities digital, it is also about creating ‘contexts for authentic learning that use new technologies in integrated and meaningful ways to enhance the production of knowledge and the communication and dissemination of ideas’ [13].

Obviously, with regard to integrating technology into the classroom setting, it is the teacher’s main responsibility to facilitate this educational innovation [14,15]. In this light, Fullan [16] formulated three important dimensions for educational innovation: (1) the possible use of adaptive material; (2) the possible use of new teaching approaches and, (3) the possible change of beliefs. While the need to investigate perceptions is emphasized by numerous authors [15,16] who stress that cognisance of end users’ perceptions of this technological innovation is crucial for predicting the success, speed and extent of its integration in classroom practice, teachers’ beliefs and attitudes towards innovation should be examined. Furthermore, research of Fullan [16] and Niederhauser & Stoddart [17] show that teachers’ beliefs are crucial; their beliefs are related to the actual uses of the implemented technology. The personal willingness of teachers to adopt and integrate innovations into their classroom practice is the key for successfull innovation [18, 19, 20]. In this context, Niederhauser et al. [17] and Becker et al. [21] distinguished between two kinds of teachers; those who either have a constructivist approach or have a more behaviourist approach to the use of technology in education. In particular, teachers who held more traditional beliefs about teaching and learning tended to use didactic instructional methods while teachers with more constructivist beliefs tended to use student-centered inquiry based methods. Understanding their beliefs is clearly a first step in the development of a deeper understanding of educational innovations in the context of complex classroom practices [22].

Integrating innovative technology during classroom practices inevitably demands teachers to acquire new technological and pedagogical skills [7]. The didactical use of tablet devices is decisive for the learning process [23]. Teachers need skills to be able to transform the learning content, the so-called Technological Pedagogical Content knowledge (TPACK) [24]. However, it is known that most of the teachers integrate technology in order to provide content in a digital way, instead of using them to enhance learner-centered approaches [23]. In this light, Welliver' s instructional transformation model [25] can be introduced. This model describes the stages that reflect the level of technology integration among teachers. The five stages are familiarization, utilization, integration, reorientation, and (r) evolution. While familiarization entails the stage where the teacher becomes aware of technology and its potential uses, (r)evolution refers to the stage where technology is a learning tool that is seamlessly woven into the teaching and learning process. In other words, technology can be integrated from *enhancing* learning to *transforming* learning.

While an amount of research described teachers’ perceptions towards a number of technological tools, such as learning management systems [26], digital learning environments [27], and blogs [28], studies concerning teachers’ perceptions of tablet computers are limited [29, 30]. Although there is relatively little academic research on the use of tablet devices, the exceptions include for instance the work of Burden [31], Clark & Luckin [7], Clarke & Svanaes [32], Montrieux et al. [30], Cumming et al. [33], and Heinrich [34]. This available perceptions-based research showed that teachers expressed positive perceptions towards using tablets [8, 30, 31, 35]. The reported instrumental advantages of this innovation were related to administrative tasks, but more importantly it enabled teachers to provide a wider range of learning activities. The availability of a wide range of apps, the connectivity to the outside world beyond the formal school walls, the immediacy of communication tools enables teachers to explore alternative activities such as 3D, multimodal, virtual tours, … In addition, teachers reported more possibilities to differentiate learning more easily and sharing information [4, 8, 33]. Cumming, Strnadova, & Singh [33] claim that teachers pointed to the possibility of ‘real world teaching’, fostering students’ involvement. Heinrich [34] pointed to the same findings concerning teacher perceptions on tablet use, involving using tablets to administrating as taking presence registrations and planning classes. They also felt that tablets promote differentiated learning. Nevertheless, implementing tablet devices into the classroom is not easy, and requires teacher training [8, 23, 31,35].

Besides the importance of the teacher, few studies examined the impact of important school-level variables that can influence the integration of technology into classes [36]. Studies of Goodison [37] and Hayes [38] have shown that local school conditions affect the integration of technology into teaching and learning practices. In particular, the importance of the ICT-coordinator, who can guide the technology implementation by scaffolding teachers [39], adequate school ICT-support [40] and school ICT infrastructure [4, 41] need to be considered during technology innovation into classrooms.

A comprehensive model that highlights the different aspects of technology integration is called the e-capacity model [42]. In this model, ICT as a lever for educational change is influenced by the teacher who has to implement technology in the classroom and can implement technology for different purposes (as a basic ICT-skills tool, a learning tool, a information tool). Followed by the actual use of the teacher, ICT-related teacher conditions are essential, such as teacher competence to deal with the technology and professional development. Next, ICT-related school conditions are needed; such as ICT-support, ICT-coordinators and ICT-infrastructure. Finally, school improvement conditions such as leadership, and the relation between teachers, and participative-decision making are relevant.

Besides the importance of measuring teacher’ perceptions towards the implementation of tablet computers, and the necessary school-supported conditions, the perspective of secondary school learners remains under-researched [43]. It is not immediately clear whether the use of technology in the classroom improves learning practices [44]. Research on the impact of tablet devices on student learning indicates that such devices have the potential to support learners by offering them a context in which they can construct and share knowledge in media-rich and stimulating environments [5]. Additionally, results suggest that the use of tablet devices seems to have an impact on student motivation and promotes meaningful learning experiences [35,45]. For instance, multiple pathways are now available for learning purposes in the form of multimedia, educational games, translation tools, and applications that support learning [7].

However, more research is needed to understand students’ perceptions concerning the use of tablet devices in an educational context [46]. The little available research, such as from Rossing, Miller, Cecil, & Stamper [47], shows that students were very positive concerning the use of tablets in higher education, stating these devices are beneficial for immediate access to information and enhancing learning experiences, according different learning styles and preferences. Other available research such as the report from Clark & Luckin [7] confirm these findings by stating students are generally reported to be positive about the tablet implementation, reporting the possibilities to motivate, engage them to learning, the possibility to make communication between peers and peers, and peers and teachers easier, and the added value for collaboration. These findings show an increased motivation, enthusiasm, interest, engagement, creativity and so on. Overall, research focusing on teachers’ and students’ perceptions towards the use of tablet devices shows that learning appears to have become more attractive. The ease of use, the availability and direct access to the World Wide Web and the lower threshold between students and teachers at the level of communication are some of the elements that reflect the changing nature of learning [7,31].

However, while recent research stated that adding 21^st^ century technologies to 20^th^ century teaching practices would just dilute the effectiveness of teaching [48]. Specific research is needed to unravel the perceptions of both teachers and students concerning the actual use of tablet devices in education. Most of the little available perceptions-based research reports on the students and teachers’ perceptions towards the impact of the tablet devices for a relatively short time period and are particularly conducted in the context of primary or higher education. Most of this kind of research reports the perceptions towards the introduction of tablet devices, where a novelty effect can partly explain the positive reactions of both teachers and students. No solid information is yet available in the literature concerning the overall perception of tablet devices. In conclusion, more research is needed to unpack the potential of tablet devices that goes further than the sales hype [6].

In order to expand existing research, a focus group study was conducted to investigate the perceptions of students and teachers on these devices after a six-month utilization period in the first ‘iPad-school’ in Belgium that has implemented tablet devices in a radical, school-wide way where both teachers and students used the tablet devices intensively during all courses, and both at school and at home. This implementation gave researchers the opportunity to investigate the students’ and teachers’ perceptions of on the changing teaching and learning practices, and as such, contribute to the existing literature. Due to the importance of gathering the perceptions towards this long-term implementation of tablet devices and the given context where we have this first iPadschool in Flanders, an explorative study was obtained. This study has the purpose to investigate the first impressions of the teachers and students towards this implementation. In this case, a explorative study was appropriate.

In order to answer the above mentioned problem statement focusing on the impact of tablet devices towards teaching and learning practices, the present study explores the following questions:

1. How do teachers and students experience the role of the teacher following the introduction of tablet devices in the classroom setting? What are, in other words, the consequences for teaching practices?
2. What are the perceptions of both teachers and students towards this change in learning practices?
3. What are the perceptions on the conditions that support these teaching and learning practices?

## Method

### Context

A qualitative focus group study was conducted in the first secondary school in Flanders (the Dutch-speaking region of Belgium) that has implemented tablet devices (iPads) into the whole school and classroom organisation. At the beginning of the school year 2012, each teacher and student received a personal tablet for use both at school and at home. Following Rogers’ [49] innovation diffusion theory, which describes five stages in the life cycle of any innovation, this school can be considered as an ‘innovator’ with regard to the introduction of tablet devices. The school can be considered a pioneer in this respect, as it was a school-wide decision to use these devices in all grades and classrooms. As this school is unique in Belgium, being the first to mobilise this education al overhaul, the appropriate approach was to set up a focus group study to examine in-depth the perceptions of both students and teachers about the introduction of tablet devices during teaching and learning practices. Conducting focus group studies gave participants for the first time after the implementation the opportunity to give their opinion and to discuss about their views. The use of focus group interviews is a common method for gathering data in qualitative educational research and was an appropriate method to gather explorative information on the perceptions of teachers and students currently using tablet computers in the pioneer school [50,51]. In particular, issues can be examined in depth. Krueger & Morgan [52] define this method as: *“A carefully planned series of discussions designed to obtain perceptions on a defined area of interest in a permissive*, *non-threatening environment”*.

### Sampling and Research Procedure

In accordance with Krueger & Casey’s guidelines [53], six focus group interviews of each ten participants were organised. More concretely, the recruitment of the participants was made ad random. Forty students and twenty teachers were invited after six months of using tablet devices in the classroom organisation. In order to select participants with different opinions, we have chosen not to accept voluntarily participants. Initially, twenty boys and twenty girls of age 11–14 and age 15–18 were selected of the student list and were invited to join a focus group interview. In particular, 20 boys and 19 girls of the 40 invited students attended the interviews. Due to practical issues, the school board selected two times ten teachers who met the proposed criteria of equal gender, more-less years of experience and the course they give (a variety of courses). In the end, only two of the twenty teachers did not participate. In sum, of the focus groups, two comprised teachers (n = 18); two comprised students aged between 11–14 years (n = 20); and two comprised students aged between 15–18 (n = 19).

For every focus group, a semi-structured interview of 90 minutes was conducted in order to investigate the participants’ initial perceptions after six months of using tablet devices. More specifically, explorative questions were structured into four categories: introduction questions, questions concerning teaching practices, questions concerning learning practices and final questions. Teachers as well as students were asked the same basic questions (see Table 1). These questions are partly inspired by the findings of Hattie [8] who claimed that the following conditions should be fulfilled in order to integrate technology into the classroom; namely the role of the teacher (research question 1), the need of professionalization (research question 3), and the need of adapted teaching and learning approaches (research question 1 and 2). Finally, the e-capacity model of Vanderlinde & van Braak [42], which focuses on the role of teachers (research question 1) and school-conditions (research question 3), was taken into account during the development of this focus group study. This model was elaborated by adding the learning aspect (research question 2) and students’ perceptions.

**Table 1:** Semi-structured interview of students and teachers.

	Questions
Introduction questions (n = 3)	‘Introduce yourself’
	‘Sum up pros and cons about using the tablet device during the courses’
	React on the statement: ‘Using tablet devices during the courses has an added value’
Teaching practices (n = 3)	‘How do you experience the role of your teachers in the classroom?’ / ‘How did you experience your role as a teacher in the classroom?’
	‘How are the courses organized?’
	React on the statement: ‘Teachers have adequate skills to teach with tablet devices’
Learning practices (n = 4)	React on the statement: ‘By using tablet devices, I achieve better’ / ‘By using tablet devices, the students achieve better
	‘React on the statement: ‘By using tablet devices, it is easier for the students to catch up the lessons’
	React on the statement: ‘By using tablet devices, it is fun to learn’
	React on the statement: ‘By using tablet devices, we work more collaboratively with peers’/ ‘Because we use tablet devices, the students work more collaboratively with peers’
Final questions (n = 3)	‘Did some problems occurred by using tablet devices?’
	‘What are your expectations for the future?’
	React on the statement: ‘Tablet devices should be introduced in every school’

Based on the three research questions, the information gathered in the teaching practices section of the interview can be used to answer the first research question. In addition, information in the learning practices section was linked to the second research question. Finally, information gathered in the introduction and final questions were related to the third research question. In other words, by conducting this focus group study, we want to examine both teachers and students perceptions towards the tablet implementation, and this with a focus on the role of the teacher and teaching practices, the impact on learning practices and with the ICT-related teacher conditions and school conditions that are needed to implement tablet devices adequately.

Concerning the role of the researchers, they took a neutral role and acted as the moderators of the conversations. To encourage conversation and integrate the tablet device into the study, the application *Socrative*, a student response system, is been used. Participants could vote and respond to statements. When sufficient data was gathered via the tablet device, a discussion took place. All focus group interviews were videotaped, and all conversations were transcribed.

### Measurements and Analysis

The transcribed conversations, the sources of this research, were loaded into the software ‘*NVivo*’, a qualitative data analysis computer software package for which a coding scheme was developed with a particular focus on three proposed research questions. Every respondent received a personal code, which was used in the coding system of *NVivo*. Based on the three proposed research questions, three coding themes were selected. Data focusing on the teaching aspect were selected under one theme, whereas data concerning the second research question (the learning aspect) are selected for the second theme. Finally, the same procedure for gathering information was used whereby the third coding theme included data that focuses on the conditions needed for implementing tablet devices into classes.

The data were analysed using a two-step procedure [54]. In the first phase of data analysis, all coded data from each focus group study was brought together and **a vertical analysis** or within-case analysis [54] was applied. This led to the creation of a case specific report which organizes and presents both the statistical and interpretative data of each focus group study in the same format using a fixed set of paragraphs. In the second data analytic phase, the results of the vertical analysis of each focus group study were submitted to a **horizontal analysis** or cross-site analysis [54] in which the six focus group studies were systematically compared for similarities and differences. To safeguard the quality of the interpretative data in both the vertical and horizontal analyses, the interpreted results were presented to the teachers involved to allow for feedback. Within this study, the entered answers in the application *Socrative* were triangulated with the focus group interview data to support the trustworthiness of the explanations of the research results.

### Ethics Statement

This research is in line with the general ethical protocol for scientific research of the Ghent University. According to their rules no additional requests to the ethical committee Psychology and Educational Sciences for advice were needed because this research project did not contain a medical bias. This study is conducted in a school, where the decision to introduce tablets into the classroom was made independent of this research. According to a signed agreement between the school and the parents or the guardians, as is stipulated in the school regulations, the host institution is responsible for the participants and has the right and the duty to end any participation in the minor’s interest. In addition to this informed consent between the school and the parents, we sent an information letter to all participants. All relevant information about this study has been adequately explained. In this information letter, we offered an opt-out choice for involvement in this study, if they would disagree with the setup of this study. We declare that this is an independent research in which the authors did not received funding from commercial institutions. According to the ethical scientific research guidelines of the Ethics Committee of the Ghent University, all data were stored anonymously. The authors have no potential conflict of interest.

## Results

The results are reported following the three research questions.

### RQ 1: How do teachers and students experience the role of the teacher following the introduction of tablet devices in the classroom setting? What are, in other words, the consequences for teaching practices?

To answer the first research question, both students and teachers described two kinds of teaching styles that occurred during the implementation process of the tablet devices, these teaching styles were labelled by the researchers as “instrumental teachers” (67%) and “innovative teachers” (33%). The results indicate that teachers’ perceptions of the tablet devices have an impact on their teaching practices.

**Fig 1:**
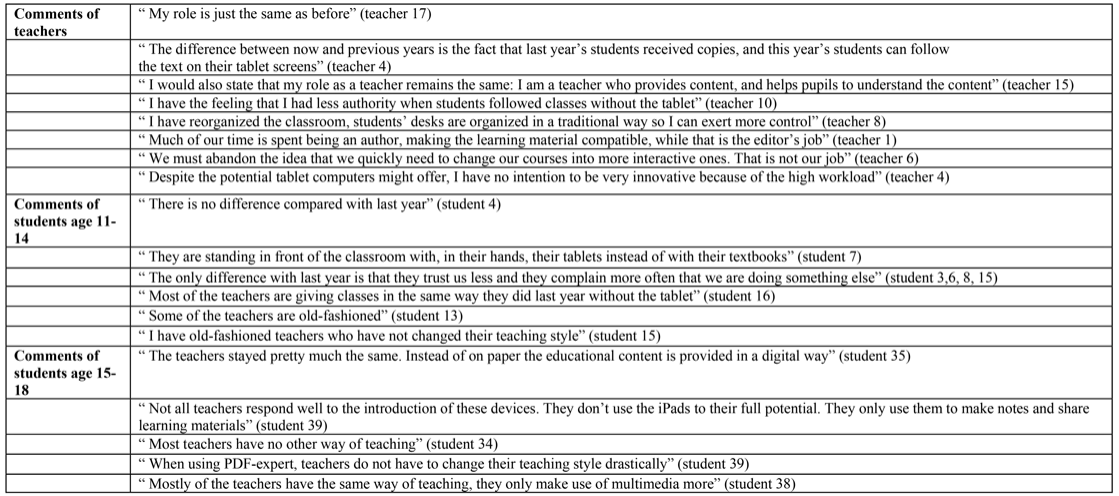
Examples of comments by teachers (n = 18) and students (n = 39) about instrumental teachers.

The “instrumental teachers” are defined by the researchers and described by the participants as those who did not change their beliefs about their role after the implementation of the tablet in their classroom and consequently, did not change their teaching style drastically. Moreover, they believe the device has a purely instrumental value since there is no need to reserve the computer lab or to copy additional work sheets. These teachers continue to accomplish their role in the same way as before, by stating that the only difference being the replacement of the textbook by the tablet. In sum, these teachers use tablet devices for teaching and learning as the participants have labelled: *“a book behind glass”*. All students state that, apart from using text-processing applications, such as PDF Expert, little time is spent on didactical applications. In addition, both the students and the teachers claim that the latter have begun to take more control in the classroom. Teachers seem to fear losing control over the class due to the presence of tablet devices, as these teachers believe students are tempted to surf social network sites and play games. Some teachers indicated they experienced difficulty dealing with the shift from their central place, in front of the classroom, to a role in which they had a less controlling function. In other words, and surprisingly, the analysis of the data showed that implementing innovative technology prompted teachers with an instrumental role to become more conservative. This instrumental behaviour could be explained in many ways. First of all, most of these teachers stated that they are disillusioned by the lack of material because many editors cannot follow or provide adapted digital material, causing teachers to feel pressured into taking on the role of authors. Secondly, some teachers mentioned the high workload, due to the lack of adequate material and the rapidness of the implementation. Thirdly, instrumental teachers generally have a traditional view on education in which they have a central role in the distribution of knowledge. In addition, due to the novelty of the tablet implementation and the school being pioneer, teachers stipulate that not many professional courses can be followed. Consequently, these teachers keep using tablet devices in a traditional way. Fourthly, these teachers claimed many students are distracted the Internet which was easier accessible. Finally, some teachers are uncertain as to precisely how the tablet devices have an added value. As a consequence, “instrumental teachers” do not seem to have the desire or energy to create innovative courses. In Fig 1, a number of comments are tabulated to illustrate these findings about the “instrumental teachers”.

**Fig 2:**
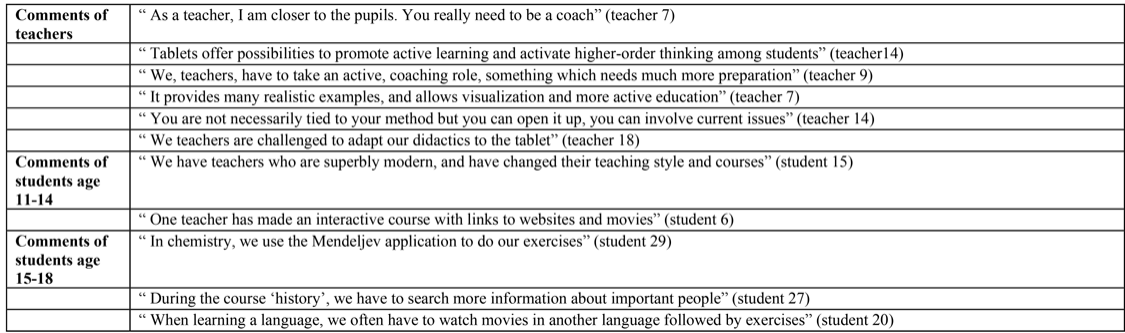
Examples of comments by teachers (n = 18) and students (n = 39) about innovative teachers.

By contrast, and illustrated in Fig 2, the “innovative teachers” are those who have adopted the role of coach. Both teachers and students indicated that using tablet devices had changed the teaching style and a wider range of learning activities could be organised (e.g. integrating multimedia and developing interactive exercises). These teachers like using more didactical applications next to text-processing ones and emphasize the need to reflect on the link between the purpose of the lesson and the specific application. Teaching and learning should be understood as processes of active learning through applications that mobilise students’ learning processes (i.e., applications to practise content or search the Internet for information). They reported that they realise that learning through the didactical use of tablets has meant a shift from traditional, teacher-centred education to the individual use of tablet devices by the students. They also believe that there is a need for a different didactics, or as the researchers call, “digital didactics”, namely content should be offered via the devices. The so-called ‘innovative teachers’ have abandoned the traditional idea that learning occurs by means of a teacher who fills minds of a passive learner with the aid of the black board. They have a different view on the learning process; and state that learning though tablet devices should allow students to negotiate the content instead of simply aiding the students to learn facts by rote. The teachers claim that these tools facilitates higher-order thinking and reasoning, and that realistic lessons can be offered in order for students to learn in an active manner. In other words, the data indicates that the innovative behaviour of these teachers is associated with a need to rethink didactical practices. Such teachers argue that their changing role makes teaching quite exhausting on the one hand, but more interesting on the other. Whereas course preparation is more intensive and puts the teachers under much pressure, teachers stipulate that it is an investment for the future. Students will learn how to deal with mobile technology in a responsible way, which is an important skill in our modern technological era. The teachers note the importance of preparing their students for a rapidly changing world, where technology is an integral part of society. These teachers believe that introducing tablet computers in secondary education is a good way to start this process as it facilitates a shift towards an active student-centred learning practice. In the end, these teachers were proud to be working for one of the first schools in Belgium to attempt this ‘new’ kind of learning, distinct from traditional learning.

### RQ 2: What are the perceptions of both teachers and students towards this change in learning practices?

**Fig 3:**
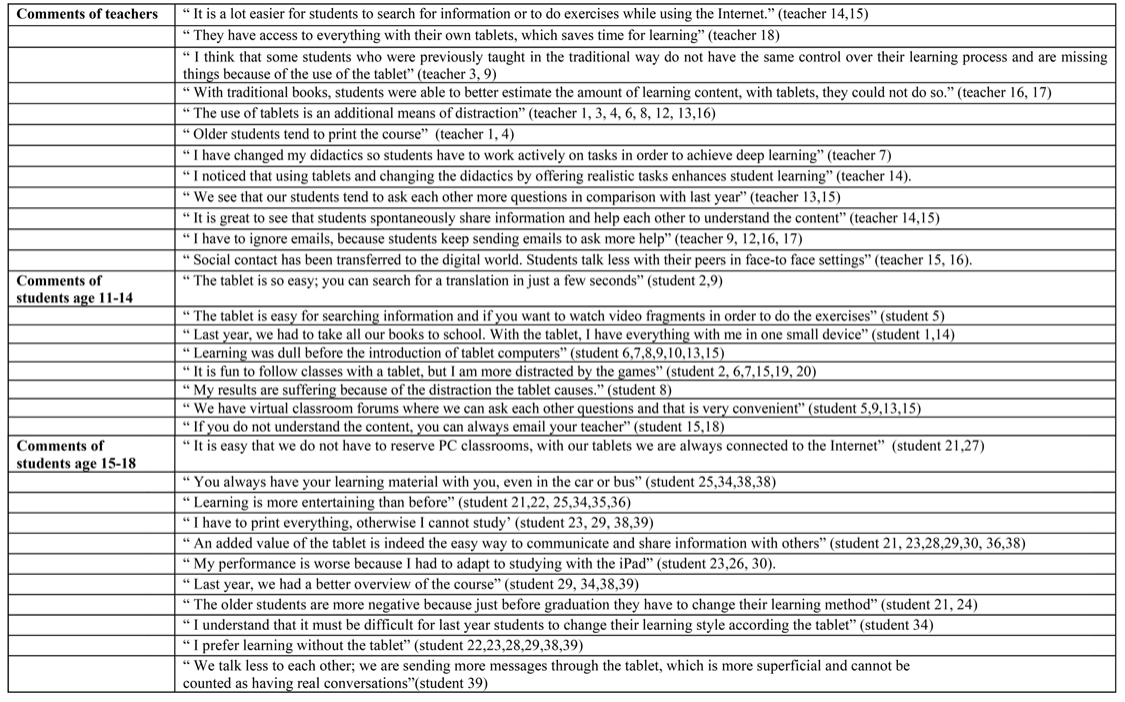
Examples of comments by teachers (n = 18) and students (n = 39) about learning practices.

In terms of the second research question, both teachers and students appreciate the added value of tablet devices, referring to the ease of use, the speed of accessing different learning materials, the ability to be able to instantly search additional information, the ability to take pictures and to integrate notes, and the reduction in the weight of their previously heavy schoolbags. These are all instrumental benefits of these new learning practices researchers label as an *‘all-in-one device’*. Moreover, as reported by most of the students, tablet devices makes learning more interesting. However, data analyses show that especially the younger students (age 11–14 years) are more positive about the use of tablet devices in school. In particular, based on *Socrative* data, 14 of the 20 students of this age reported that learning was dull before the introduction of tablet computers. In contrast, most of the older students tend to print the course material, use keyboards for writing and are more critical about the use of tablets for learning. More specifically, 13 of the 19 students of this age stated that aside from the added value of using the tables to access multimedia, their overall learning capacity has not increased. These students indicated the problem of having less of an overview of the course content and the major issue of distraction. The teachers also mentioned these disadvantages as a concern. As a distinction can be made among instrumental and innovative teachers, it can concluded that older students tend to have a classical view about learning practices, in comparison with the younger students. However, it seems that the role teachers take also has an impact on learning practices. Both students and teachers stated that if teachers integrate the tablet in a meaningful manner (adopt the role of an innovative teacher), deep learning could be achieved. Innovative teachers stated that learning through these devices could open many doors; one can integrate elements of the daily lives of students, which promotes meaningful learning. The introduction of tablet devices has facilitated a shift from ‘learning by heart’ to learning through multimedia, social media and the integration of the personal world of children, all of which makes learning more meaningful. Finally, as reported by almost every student and teacher, a large benefit of using tablet devices for learning is that students seem to be more inclined to collaborate and share information with each other. Both teachers and students (age 11–18) stated that by using applications such as iMessage and Facebook, students establish online networks and discussion groups spontaneously. Furthermore, more than 75% of the students indicated that the ability to ask their peers questions aids their learning process. Nonetheless, a small number of teachers and students have stated they have concerns about this transfer of social contact to the digital world. Apart from the enhancement of communication between peers mutually, communication between teachers and students is more frequently reported. In addition, one of the central issues of tablets for students’ learning is the blurring of the boundary between school and recreation. Participants claim that the traditional context of learning between the school walls has now extended to a digital learning environment, which allows children and teenagers to access content anywhere and at any time. This shift has both positive and negative aspects. While some participants indicated the benefit of this ‘extended’ kind of learning (i.e., learning that is not limited to the physical school context), others experienced difficulties with this blurred boundary. Additionally, some teachers questioned the need to answer emails from students after school hours, while some students emphasised the temptation to play games or chat with friends when they should be studying. In summary, see Fig 3 for some examples of comments on learning practices.

### RQ 3: What are the perceptions on the conditions that support these teaching and learning practices?

With reference to the final research question, the results of the focus group interviews revealed that further support is needed for teaching and learning practices. This need is categorized by the researchers into two preconditions, namely material conditions and the need for professionalization, and should be taken into account when integrating tablet devices in schools.

**Fig 4:**
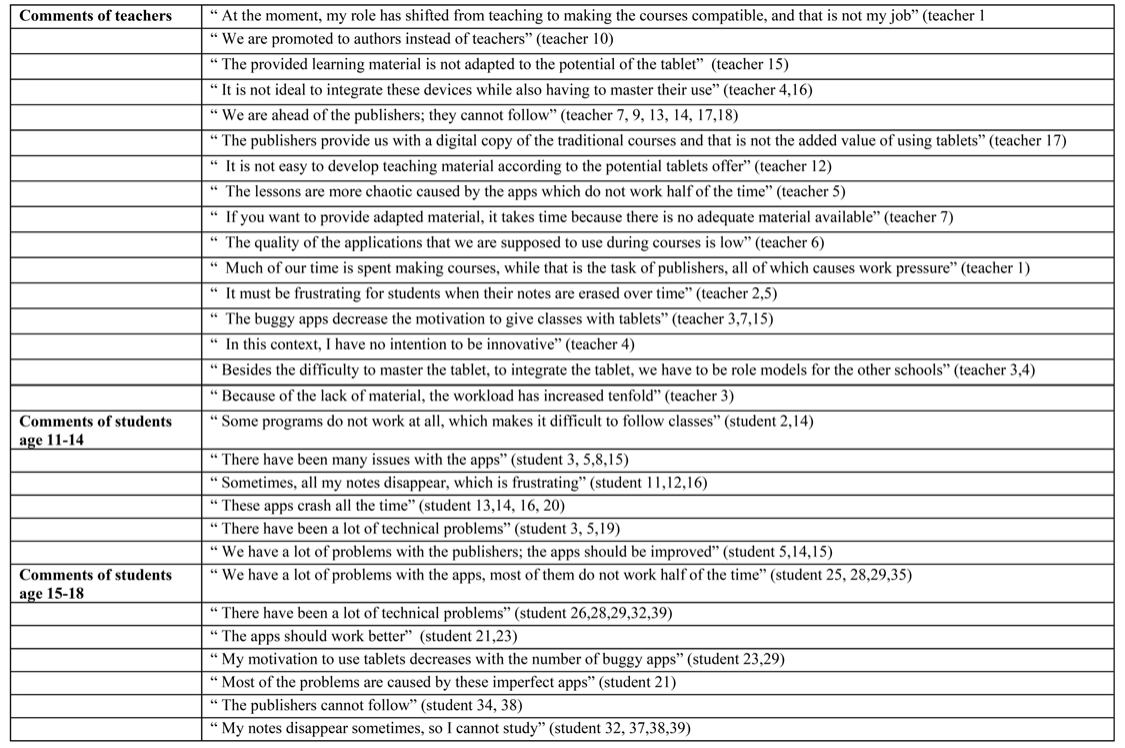
Examples of comments by teachers (n = 18) and students (n = 39) about the conditions that support teaching and learning practices.

First, as already mentioned in the first research question and as illustrated in Fig 4, it is clear that most of the interview time was spent to discuss the needed support and the obvious lack of adequate teaching material using the potential and design of the tablet devices. Teachers and students report the feeling of being somewhat abandoned by publishers, who are lagging behind and provide, as the researchers call, *“book behind glass”* material instead of adapted teaching materials. In this context, teachers who have not yet taken the innovative teaching role are somewhat thwarted. If editors cannot provide adapted digital material, teachers stated that they are pressured into taking on the role of the actual authors, which leads to high workloads and overall sceptical attitude towards of the integration of tablet devices into their courses. This attitude seems to be one of the most important obstacles preventing the adequate integration of the tablet device. Aside from inadequate teaching materials, the teachers pointed to the speed of the school-wide implementation of the devices, which has also caused teachers to experience an increased workload. It was a rather ‘provocative’ choice of the school board to implement the tablet on the first day of the school year, without knowledge as to whether publishers would follow. With such an abrupt change, not every teacher was given the time to master the use of the device itself and some were unable to find a balance between teaching with devices and being role models for the outside world. Secondly, using tablet devices requires a fast Internet connection and if problems occur, the lesson flow is disrupted. In the interviews, technical and Internet problems were reported to have occurred during classes, which was disillusioning for both students and teachers. Commonly occurring problems included websites that would not open or applications that crashed. However, as students have easy access to the Internet, many teachers complained their students were playing online games.

**Fig 5:**
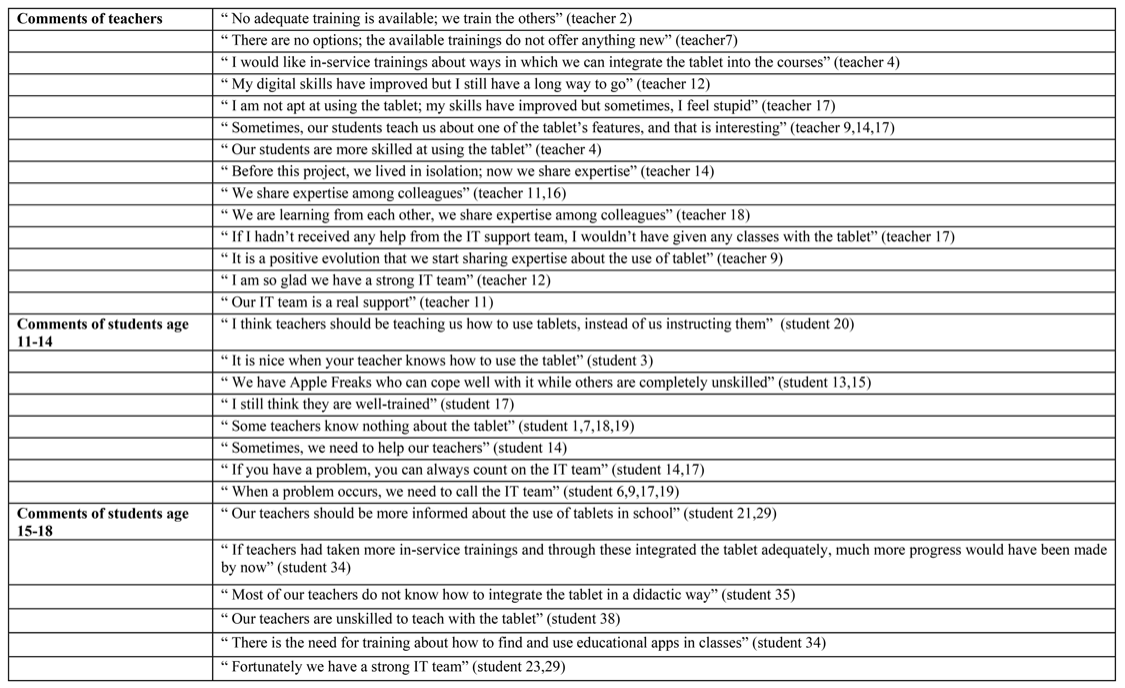
Examples of comments by teachers (n = 18) and students (n = 39) about the condition professionalization.

The final supporting condition is the need for professionalization. As depicted in Fig 5, the results show that while students aged between 11–14 years are indifferent to their teachers’ need for professional development, the older students have become sceptical about the digital competence of their teachers. These students believe that the teachers need to attend training courses including information about techniques and applications that promote interactive learning. In other words, teachers need to develop adequate skills so that the course content can be optimally delivered via tablet devices.

As depicted in Fig 5, the teachers expressed mixed opinions concerning their need for professional development. Innovative teachers deem themselves to be skilled tablet device users and state that is their responsibility to provide training and share their expertise with other schools. Concerning the need of training, instrumental teachers expressed two views. While some felt the need for additional instruction, others felt that they simply had no energy left and had thus no desire for additional training. Apart from the need for training, both students and teachers indicate that a strong and competent IT team would contribute to the success of this academic innovation.

## Discussion and Conclusion

This study contributes to the existing literature in several respects.

First, the goal of this study was to unravel the perceptions of important stakeholders on the use of tablet devices in secondary education. While investigating perceptions is necessary to analyse the use of technological innovations in education [15,16], specific research that goes further than relatively short-time research, including the influence of the novelty effect when introducing tablet devices, is lacking. Besides the need of investigating teacher perceptions [14,15]; the perspective of secondary school learners remains under-researched [43,46]. More research is needed to unpack the potential of these devices, more specifically research that goes further than the sales hype [6]. Thus, examining the perceptions of both teachers and students after a considerable time of intensive tablet use is desirable. In order to elaborate existing research, an explorative focus group study was conducted in the first iPad-school in Flanders where the tablet is used in daily teaching and learning practices.

As the results for the first research question have shown, this study confirms that perceptions on any educational innovation will influence its practical integration [10, 16, 17], and that teachers are key to the success of the implementation of technology in schools [14, 19,20]. The results for the first research question are in line with previous research of Becker et al. [21] and Niederhauser et al. [17], who also mentioned that teachers could be distinguished in two groups, namely the behaviourist teachers and the constructivist teachers. Indeed, the results of current study indicate that the teachers using tablet devices can be distinguished into two categories, which has clear consequences for teaching and learning practices. In this context, we labelled the “constructivist teachers” as “innovative teachers”. Innovative teachers attempt to shift from a teacher-centred to a learning-centred innovative approach, integrating educational applications during courses. The “behaviourist teachers” are defined in this study as “instrumental teachers”. Instrumental teacher are teachers who view the instrumental benefits of the tablet and appear to maintain the traditional way of teaching, even while using the tablet devices, since they essentially end up with a simple *“book behind glass”*. In addition, these two types of teachers can be related to Welliver' s instructional transformation model [25]. Instrumental teachers can be linked to teachers, who stuck in using tablets without a fundamental change in teaching and learning approaches, who use technology as a functional improvement to *enhance learning*. Innovative teachers can be linked to the highest level of technology integration whereas teachers use tablet devices to *transform learning*, which opens teaching and learning practices which were previously inconceivable.

Taken together, the results show that the introduction of innovative technology seems to provoke conservative practices among teachers with an instrumental view as they adopt a stringent role and give traditional courses with a tablet device. This is somewhat contrary to the overall intention of introducing tablet devices; instead of revolutionising education, to the tablet strengthened the old educational structures. In other words, to conduct a lesson in a traditional way with tablet devices consolidates the ‘ancient’ didactic model. This consolidation underlines the importance of switching to digital didactics where the tablet device is integrated in such a way that it is used to its full potential during lessons.

Secondly, findings concerning the second research question of this study corroborate the educational potential of these devices, such as browsing the Internet, working together spontaneously, and using multimedia for a better understanding the course content. Results confirm that tablet devices comprise learning activities that were previously not possible [7,31, 33], making the device an asset to the learning process. Students in this study reported that in particular the innovative teachers use tablet devices to provide authentic learning experiences and where they can construct and share knowledge in a media-rich environment, which is in line with the little available research [5,7,35,45]. However, both the students and (innovative) teachers also remarked that the introduction of tablet devices entails a shift in learning, for which not all students are ready. Younger students appear to be more flexible in respect to learning through devices, while it seems difficult for older students to change their study habits. This opens avenues for further research.

Thirdly, the fact that the older students in this study advise teachers to improve their didactical skills in order to master tablet devices is a new phenomenon in the research literature. This finding highlights the importance of taking into account the perceptions of students when investigating the introduction of new technology into education [43].

Fourthly, following Hattie [8] and the e-capacity framework of Vanderlinde & van Braak [42], and as an answer to the third research question, more attention should be paid to the preconditions that ensure the development of innovative teachers through the provision of adapted learning material and an adequate IT infrastructure. Most teachers reported feeling abandoned by publishers due to the lack of adequate teaching material appropriate for the tablet devices. If editors cannot follow or provide adapted digital material, teachers are under pressure to adopt the role of academic authors, which, in turn, generates a heavy workload and ambivalence about the integration of tablet devices into courses. Policy makers should take into account the lack of adequate teaching materials because this deficiency will be an obstacle for schools to implement this technology successfully. Hence, publishers also have a certain responsibility in facilitating the success of these educational innovations. Without appropriate equipment, many teachers will simply use the device in an instrumental way.

Fifthly, more attention should be paid to the (formal and informal) professional development of teachers to support them in this educational reformation [31]. This finding can be linked to the e-capacity framework of Vanderlinde and van Braak [42], describing that educational change depends on important school-level variables such as an adequate ICT-support team, infrastructure and teacher professional courses. Introducing new technology into education generates a simultaneous need for professionalization. As indicated above, the teacher’s role and competences are crucial to the success of this innovation; adapted teaching materials and equipment are essential in this respect. Aside from professionalization and the need for adapted teaching materials, teachers need time to become familiar with these new devices. To conclude, this study shows that certain supporting conditions must be in place when implementing technological educational tools [7]. Moreover, an educational policy that provides adequate preparation for teachers, attention to digital didactics, adapted didactical material, and technical and pedagogical support will stimulate teacher and student recognition of tablet devices’ potential in education. In sum, policy makers and educators should take the possible pitfalls into account mentioned in this study so lessons can be drawn in order to ensure the smoother future implementation of this technology. Avoiding such pitfalls will facilitate the development of new teaching and learning practices in accordance with the needs of the current digital age.

## Limitations and Further Research

Apart from qualitative research being an excellent method to gather information about perceptions in depth, some limitations could be formulated. For example, data are collected from a few individual cases and based on first-person perspectives. However, we believe that these findings have an added value to the research world in several respects and that they can be transferable to other innovative settings. In addition, only perceptions of the participants are examined in this study, while these results challenge teaching practices and the development of learning materials in future research it would be interesting to investigate the impact of such tablets on teaching and learning practices. Additional empirical research is needed to gain further insight into the impact of modern technology on teaching and learning. Future research will be conducted to answer didactic questions such as: how should tablet devices be implemented in class contexts and does the use of tablet computers increase motivation and achievement among students? Research on this topic is still an unexplored area, requiring further in-depth studies.
